# Alzheimer’s Disease: Biomarkers in the Genome, Blood, and Cerebrospinal Fluid

**DOI:** 10.3389/fneur.2017.00102

**Published:** 2017-03-20

**Authors:** Rose Ann Huynh, Chandra Mohan

**Affiliations:** ^1^Department of Biomedical Engineering, University of Houston, Houston, TX, USA

**Keywords:** Alzheimer’s disease, early detection, genetic biomarkers, neurochemical biomarkers, blood-derived biomarkers, longitudinal studies

## Abstract

Alzheimer’s disease (AD) is a progressive neurodegenerative disorder that slowly destroys memory and thinking skills, resulting in behavioral changes. It is estimated that nearly 36 million are affected globally with numbers reaching 115 million by 2050. AD can only be definitively diagnosed at autopsy since its manifestations of senile plaques and neurofibrillary tangles throughout the brain cannot yet be fully captured with current imaging technologies. Current AD therapeutics have also been suboptimal. Besides identifying markers that distinguish AD from controls, there has been a recent drive to identify better biomarkers that can predict the rates of cognitive decline and neocortical amyloid burden in those who exhibit preclinical, prodromal, or clinical AD. This review covers biomarkers of three main types: genes, cerebrospinal fluid-derived, and blood-derived biomarkers. Looking ahead, cutting-edge OMICs technologies, including proteomics and metabolomics, ought to be fully tapped in order to mine even better biomarkers for AD that are more predictive.

## Introduction

Alzheimer’s disease (AD) is a progressive neurodegenerative disorder that slowly destroys memory and thinking skills, resulting in behavioral changes. This places an emotional toll on the patients’ caretakers, while placing a financial burden of approximately $214 billion in 2014 alone on the American society (1). The number of those affected is expected to reach 115 million worldwide by 2050. Thus, there is an ever-growing need to find biomarkers for the early diagnosis of AD as well as to predict disease progression. AD is defined by the presence of senile plaques and neurofibrillary tangles (NFTs) throughout the brain resulting in its shrinkage. Definitive AD pathology can only be determined at autopsy since these neurologic manifestations are not readily perceptible using current diagnostic technologies, making early diagnosis difficult and inaccurate (1, 2). Current scientific evidence suggests that in preclinical Alzheimer’s, brain changes caused by the disease may begin years before symptoms of mild cognitive impairment (MCI) set in Ref. (3). Also, current therapeutic approaches have been largely inadequate toward addressing the earliest clinical symptoms of AD, where neurological damage is irreversible (4). Thus, comprehensive research is being performed to identify better biomarkers for the early detection of AD with the hope that an effective therapeutic window will emerge.

As Alzheimer’s research has progressed, two clinically accepted validated biomarkers, amyloid-β (Aβ) accumulation in cerebrospinal fluid (CSF) and Pittsburgh compound B positron emission tomography (PiB-PET) measurements, have changed the primary focus of biomarker research from differentiation between cognitively normal healthy controls (HC) and AD, to the ability of potential biomarkers to predict the rates of cognitive decline in those who exhibit preclinical, prodromal, or clinical AD (3, 5–8). Previous review articles have shed light on potential biomarkers meeting some of these criteria including imaging and animal studies (9–14). In contrast to those reviews, the present article will focus on updating our current understanding of AD biomarkers harnessed from the genome, the CSF, or the blood, since there have been rapid developments in this arena; moreover, the laboratory assays for these biomarkers are relatively inexpensive (compared to neuroimaging modalities). At the close of this review, we will also discuss longitudinal studies utilizing these biomarkers, individually and in combination, to better evaluate which of these may be clinically useful as long-term markers of AD progression.

## Genetic Biomarkers

Several researchers have investigated the genetic makeup of those afflicted with Alzheimer’s compared to neurologically healthy individuals. *Amyloid-*β *precursor protein* (*A*β*PP*), *presenilin 1* (*PSEN1*), and *presenilin 2* (*PSEN2*) genes have been strongly implicated in early onset Alzheimer’s disease (EOAD), particularly familial EOAD, which comprise less than 5% of Alzheimer’s cases (15). In contrast, late onset Alzheimer’s disease (LOAD) has been associated with other genes including *apolipoprotein E-*ε*4* (*APOE* ε*4*), *bridging integrator 1* (*BIN1*) region, *clusterin* (*CLU*), *phosphatidylinositol clathrin assembly lymphoid-myeloid* (*PICALM*), and *complement receptor 1*, mostly identified through genome-wide association studies (GWAS) (http://www.alzgene.org/). Among these most highly associated genes for LOAD, the *APOE* ε*4* allele emerges as the most promising candidate. This relative rank of gene association to AD is based on the human genome epidemiology network interim criteria for the cumulative assessment of genetic associations, which takes into account the sample size, heterogeneity across studies, and protection from bias within these studies.

Genetic tests are available for both *APOE* ε*4* and the rare genes associated with EOAD. Traditionally, screening for AD associated genes *via* targeted sequencing methods, whether through Sanger or next-generation sequencing (NGS), was more commonly used than whole-exome sequencing due to lower costs and faster analysis (16). However, as the cost and running time of whole-exome sequencing has decreased, particularly through refined NGS methods, it has become a more widely used tool for genetic screening (17, 18). However, routine clinical testing for early detection is not currently recommended for most EOAD individuals since there is currently no effective treatment or prevention for AD. Moreover, the identification of a mutation is not a certain predictor of disease or onset age, given that these mutations can vary in terms of penetrance and gene expression (19). However, an increasing number of studies report the benefits of disclosing Alzheimer’s diagnosis early since it allows the patient to plan for their future with better access to good medical care and support services, as reviewed elsewhere (20). In fact, *PSEN1* and *PSEN2* testing is now recommended for individuals with early onset dementia who have at least one affected family member. *PSEN2* testing is even further recommended for those individuals who also present with delusions or hallucinations (21). Likewise, *A*β*PP* testing is now recommended for individuals with early onset dementia who have at least one affected family member and in whom no *PSEN1* mutation has been identified (21).

## APOE ε4 Allele

Among the many genes examined, the *APOE* ε*4* allele emerges as the strongest genetic risk factor for AD (22). ApoE regulates lipid homeostasis by mediating lipid transport from one tissue or cell type to another. In the central nervous system (CNS), ApoE is produced mainly by astrocytes where they transport cholesterol to neurons *via* ApoE receptors (23). In humans, *APOE* polymorphism is represented by three different alleles: ε2, ε3, and ε4, which generally occur at frequencies of 8.4, 77.9, and 13.7%, respectively, in Caucasian populations (24, 25). However, in those afflicted with AD, the frequencies are altered to 3.9, 59.4, and 36.7% for the ε2, ε3, and ε4 alleles, respectively. These frequencies vary based on ethnic groups; whereas the Japanese population exhibits the highest, the Hispanic population exhibits the lowest frequency differences of the homozygous *APOE* ε*4* allele in AD patients versus healthy controls (24, 25). This difference in AD frequencies between ethnic groups is also reflected in their respective odds ratios (ORs) for AD: whereas the Japanese population exhibits the highest OR (33.1), the Hispanic population exhibits the lowest (2.2) OR for the *APOE* ε*4* homozygous genotype (Table 1).

**Table 1:** **Comparison of *APOE* ε*4* allele as a marker for AD in various studies**.

Reference	Disease comparison (ethnic group)^a^	No. of cases (AD/non-AD)	*APOE* ε*4* allele carrier type	Specificity (95% CI)	Sensitivity (95% CI)	PPV/NPV	OR (95% CI)
Farrer et al. (24)	AD versus HC (Caucasian)	(193/6,262)	Homozygous	N/A	N/A	N/A	12.5 (8.8–17.7)
	AD versus HC (African American)	(34/240)	Homozygous	N/A	N/A	N/A	5.7 (2.3–14.1)
	AD versus HC (Hispanics)	(12/267)	Homozygous	N/A	N/A	N/A	2.2 (0.7–6.7)
	AD versus HC (Japanese)	(45/1,977)	Homozygous	N/A	N/A	N/A	33.1 (13.6–80.5)
Saunders et al. (26)	AD versus HC	(46/10)	Heterozygous	1	0.75	1.0/0.42	N/A
	AD versus HC	(11/10)	Homozygous	1	0.19	1/0.18	N/A
Elias-Sonnenschein et al. (27)	AD versus MCI	(35/35)	Heterozygous	0.67 (0.62–0.71)	0.53 (0.46–0.61)	0.43/0.75	2.29 (1.88–2.80)
	AD versus MCI	(9/9)	Homozygous	0.93 (0.90–0.97)	0.21 (0.10–0.33)	0.59/0.89	3.94 (2.09–7.33)
Vos et al. (28, 29)	AD versus naMCI	(73/226)	N/A	0.62 (0.53–0.71)	0.56 (0.37–0.74)	0.25/0.86	1.8 (0.7–4.7)
	AD versus aMCI	(158/399)	N/A	0.57 (0.49–0.64)	0.56 (0.46–0.66)	0.40/0.71	1.8 (1.1–3.0)

*AD, Alzheimer’s disease; aMCI, amnestic mild cognitive impairment; APOE, apolipoprotein E; HC, healthy control; MCI, mild cognitive impairment; naMCI, non-amnestic mild cognitive impairment; OR, odds ratio; PPV/NPV, positive predictive value/negative predictive value*.

*^a^Ethnic group included if only a particular group was studied*.

Corder et al. reported that the *APOE* ε*4* allele increases the amount of amyloid deposition in a gene dose-dependent manner, which was mirrored by their findings that AD frequency was increased among *APOE* ε*4* homozygous individuals compared to those with only one *APOE* ε*4* allele (91.3 versus 46.6%, respectively) (30). Moreover, the mean age of clinical onset of AD was accelerated in *APOE* ε*4* homozygous individuals compared to the heterozygotes (68 versus 76 years of age, respectively). For those lacking the *APOE* ε*4* gene, AD frequency was found to be 20.4% with a mean onset age of 84 years. This increase in AD frequency among *APOE* ε*4* homozygotes may be due to impaired delivery of cholesterol from astrocytes to neurons where increased cholesterol concentration, especially those found in the membrane, may induce the accumulation of amyloid-beta1-42 (Aβ_42_) through the induction of the β-secretase pathway (31). In 1996, Saunders et al. sought to quantify the diagnostic value of the *APOE* ε*4* allele in a real clinical setting (26). For both heterozygous and homozygous patients, they found that the positive predictive value (PPV) was 100%, indicating that those who tested positive for the *APOE* ε*4* allele truly had AD. However, the negative predictive value (NPV) was low for both groups indicating that the absence of the allele does not necessarily prelude AD (Table 1).

In 2011, Elias-Sonnenschein et al. performed a meta-analysis to determine the predictive value of the *APOE* ε*4* allele for progression from MCI to AD-type dementia (27). They extracted data from 35 studies involving a grand total of 1,236 AD patients. They found that the *APOE* ε*4* allele was a moderately strong predictor of progression from MCI to AD-type dementia, with a PPV of 0.59 for *APOE* ε*4* homozygotes. However, the PPV was considerably lower for the heterozygotes, and sensitivity was low for both groups (Table 1). These findings suggest that *APOE* ε*4* may have limited utility in predicting disease progression in AD. Recently, Vos et al. investigated whether potential genetic markers for AD were differentially distributed in patients with amnestic mild cognitive impairment (aMCI) versus those with non-amnestic mild cognitive impairment (naMCI) (28). They found that the *APOE* ε*4* allele was similarly prevalent in naMCI and aMCI, at 40 and 47%, respectively. This contrasted with prior studies reporting that the absence of *APOE* ε*4* allele was related to less memory impairment and should therefore occur at a much higher frequency in aMCI compared to naMCI (23). Similarly, the sensitivity and specificity of the *APOE* ε*4* allele in predicting the development of AD from naMCI and aMCI were very similar (Table 1). This may have been due to the fact that they did not distinguish *APOE* ε*4* allele carriers into homozygotes versus heterozygotes, and this becomes pertinent since the *APOE* ε*4* allele behaves in a gene dose-dependent manner (25).

## Other Potential Genetic Biomarkers

A couple of additional genetic markers have also been accorded relatively high ORs in the earlier GWAS studies (http://www.alzgene.org/), as well as a more recent meta-analysis (32), including *BIN1, CLU*, and *PICALM*. The *BIN1* variant rs6733839 (OR of 1.22; meta *p*-value of 6.9 × 10^−44^) (32) and an additional variant (rs59335482; an insertion of 3 cytosine bases) ~28 kb upstream of *BIN1* have been associated with increased AD risk (OR of 1.20; *p*-value of 3.8 × 10^−11^) (33). *BIN1* is a widely expressed adapter protein that functions in clathrin-mediated endocytosis and endocytic recycling, leading some researchers to believe that *BIN1*, in turn, functions in AβPP metabolism (34). *BIN1* is also involved in the regulation of cytoskeleton dynamics, where the tubular membrane structures it forms appear to link the microtubule skeleton with the cellular membrane, leading others to believe that it modulates tau pathology (35, 36). *PICALM* is another widely expressed gene that encodes an adapter protein that functions in clathrin-mediated endocytosis and endocytic recycling, whose polymorphic variants rs3851179 and rs10792832 have also been associated with AD in GWAS studies (32, 37, 38). Although it is associated with similar functions as *BIN1* (i.e., clathrin-mediated endocytosis and endocytic recycling), it exhibits weaker association with LOAD (OR of 0.87; meta *p*-value of 9.3 × 10^−26^ for SNP rs10792832) (32). Although the exact role of PICALM in AD pathogenesis is unclear, PICALM has been implicated in APP processing and increased Aβ production in neurons (39).

*Clusterin*, also known as apolipoprotein J, or CLU, is another gene significantly associated with LOAD, with an OR of 0.88 and a Bayes factor of 20.1 (http://www.alzgene.org/) and an OR of 0.86 and a *p*-value of 2.8 × 10^−25^ (32). This molecule is abundantly expressed in neurons and astroglia, and tends to colocalize with Aβ, particularly in senile plaques (40). It has been suggested by several studies that *CLU* plays a protective role in AD pathogenesis through the prevention of Aβ fibrillization, clearance of Aβ, inhibition of the complement system and neural apoptosis, and promotion of neurite growth (41–43). In particular, the rs11136000 and rs9331896 variants of *CLU* are associated with reduced AD frequency (OR of 0.89 and 0.86, respectively) (38). However, increased levels of CLU have been found in the CSF, frontal cortex, and hippocampus of AD patients. This apparent contradiction has led to the suggestion that some *CLU* AD-risk variant carriers may have an increased likelihood of developing AD later in life due to a diminished neuroprotective action of CLU, which may in turn lead to excessive production of CLU later in life (43). These findings are supported by a more recent study published in 2016 by Weinstein et al. suggesting that the association between plasma CLU levels and the risk of dementia may be dependent on age or an age-related factor (44). In this study, CLU was significantly associated with an increased risk of dementia among elderly subjects (>80 years of age; HR of 6.25) but reduced risk of dementia among younger subjects (<70 years of age; HR of 0.53).

Another genetic variant, only recently reported, also shows promise as a genetic marker of AD. Jonsson et al. analyzed the genomic sequences of 2,261 Icelanders and found rs75932628-T in *triggering receptor expressed on myeloid cells 2 (TREM2)* to be associated with a 2- to 4.5-fold increased risk of developing non-familial AD (45). The OR was 2.26 for all population controls, 2.92 for population controls 85 years of age or older, and 4.66 for cognitively intact controls (score of 0 on Cognitive Performance Scale) who were 85 years of age or older. The OR of rs75932628-T in the African American population was lower with a value of 1.83, suggesting that the diagnostic value of this genetic variant may be ethnicity dependent (46). Additionally, another variant of *TREM2*, rs79011726, exhibited an OR of 2.14 in the African American population. TREM2 is an immune receptor that is found in brain microglial cells (47). Although the exact link between this genetic variant and AD pathogenesis is unclear, animal studies have indicated that microglia play an important role in how the brain responds to Aβ plaques (48).

Besides the above players, a few additional genes have also been uncovered through the more recent GWAS International Genomics of Alzheimer’s Project (IGAP) (32), including an association in the *HLA-DRB5*–*DRB1* region (OR of 1.11; meta *p*-value of 2.9 × 10^−12^; SNP rs9271192). This region is involved in immunocompetence and histocompatibility and has already been shown to be a risk factor in multiple sclerosis (49). Since its discovery in 2013, one study has explored the role of *HLA-*DRB5, as well as 11 other newly discovered genetic risk factors (*PTK2B, SORL1, SLC24A4, DSG2, INPP5D, MEF2C, NME8, ZCWPW1, CELF1, FERMT2*, and *CASS4*), in AD pathogenesis. Recently, Yu et al. have reported that brain DNA methylation near *HLA-DRB5* was associated with pathological AD (*p*-value of 5.0 × 10^−5^) (50). Similar results have also been reported with some of the other novel genetic risk factors documented by IGAP, including *SORL1* and *SLC24A4*, and there will undoubtedly be more studies to come.

## Combined Genetic Risk Scores (GRSs)

Recently, multiple studies have attempted to combine different genetic risk factors to determine whether a combined genetic panel could more accurately predict AD risk. In 2015, Adams et al. reported that there was a significant interaction between their AD GRS, containing 19 genetic variants, and the age-at-onset of dementia, whereby a stronger genetic effect was noted at earlier ages (51). This AD GRS included some of the genes mentioned above, such as *APOE, PICALM, CLU*, and *BIN1*. Adams et al. did not include mutations implicated in familial AD (*PSEN1, PSEN2*, and *A*β*PP)*, since they wanted to focus on sporadic AD. They found that the novel AD-risk variants identified through GWAS were associated more closely with naMCI (OR 1.25), while *APOE* ε*4* was better associated with aMCI (1.16) (51),. These results suggest that AD genes might influence different cognitive domains, but this appears to be in conflict with the 2013 study by Vos et al. particularly for *APOE* ε*4*, where Vos et al. reported that the *APOE* ε*4* gene exhibited similar sensitivity and specificity when predicting the development of AD from naMCI and aMCI (Table 1). Nevertheless, Adams et al.’s results are in agreement with prior studies that reported that the *APOE* ε*4* gene occurs at higher frequencies in aMCI compared to naMCI (23). Though the association of the AD GRS to MCI and incident dementia is modest, the size of the study (*N* = 3,605) along with the continued follow-up of the initially non-demented subjects over 7–10 years raises hope that this GRS would be of utility in predicting disease development among cognitively normal individuals and that it may lead to a clinically useful test for AD. These findings also warrant a closer look at the effect of each individual genetic locus implicated in AD pathogenesis, particularly when preceded by MCI, a genetically heterogeneous condition.

In 2015, Sleegers et al. also sought to study the predictive value of GRSs, which included several genes already discussed in this paper: *APOE, PICALM, CLU*, and *BIN1* (52). Four different GRS models were created that are as follows: (1) Model_APOE-S_, (2) Model_APOE-A_, (3) Model_ALL-WS_, and (4) Model_ALL-WA_. The most promising and significant of these models was Model_ALL-WA_, which yielded a sensitivity of 55%, specificity of 78%, AUC of 0.70, and OR of 2.32 for discriminating AD patients, whether familial or sporadic, from healthy controls. Model_ALL-WA_ was further analyzed for its discriminative ability between familial and sporadic AD. It was found to be a stronger predictor of familial AD (OR of 3.01 versus 2.14). Logistic regression analysis of Model_ALL-WA_ also showed an increased risk of AD with increasing risk score per unit increase in GRS [OR, 2.32 (95% CI, 2.08–2.58)], with this effect being more prominent in familial AD. Interestingly, the authors found no notable differences in the genetic risk profile in the presence or absence of *APOE* ε*4* in their study population. Another recent study examined the interaction between *PICALM* and *APOE* and concluded that the PICALM genotype could modulate both brain atrophy and cognitive performance in *APOE* ε*4* carriers, and may perhaps be responsible for the absence of *APOE* ε*4*’s effect on the GRS reported by Sleegers et al. (53). Clearly, genetic interactions between the different genes implicated in AD and their functional consequences warrant further analysis.

## CSF-Derived Neurochemical Biomarkers

Sampling CSF is a relatively non-invasive method for assessing pathologic alterations occurring within the CNS (9), although, some still consider the required lumbar puncture to be invasive, as there is a small risk of bleeding or brainstem herniation. Due to the CSF’s direct contact with the CNS, CSF-derived biomarkers have been studied extensively in AD (9, 54).

## CSF Aβ Peptides

It is well established that senile amyloid plaques, composed largely of Aβ peptides, accumulate in the cerebral cortex and hippocampus during the early stages of AD (54, 55). The principal Aβ species deposited is Aβ_42_ as it is more hydrophobic and fibrillogenic. These aggregates of Aβ injure synapses, ultimately causing neurodegeneration and dementia (56). This accumulation of Aβ_42_ within the cerebral cortex is hypothesized to be the result of overproduction of Aβ_42_ and/or reduced efflux of Aβ_42_ across the blood–brain barrier into the CSF. Whatever the exact mechanism, the levels of Aβ_42_ within the CSF are decreased as a result (25). This decrease in baseline CSF levels serves as a potential basis for AD diagnosis. However, the exact baseline and cutoff value of CSF Aβ_42_ chosen for AD diagnostics has varied between different research group, hence yielding differing diagnostic performance metrics. Recent studies have begun to standardize their assay methods, resulting in more comparable results.

In 2003, Kapaki et al. evaluated the diagnostic potential of CSF Aβ_42_ as a biomarker of AD (57). They particularly sought to differentiate AD from normal aging and other non-AD neurodegenerative dementias (NAND) within the Greek population. They found a 0.5-fold decrease in CSF Aβ_42_ levels in AD patients compared with normal aging, whose baseline level was 738 pg/ml. In 2006, De Jong et al. found a similar fold difference of CSF Aβ_42_ basal levels in their AD group compared to healthy controls (58). The latter study attained a sensitivity of 0.93 compared to 0.82 in Kapaki et al.’s study, which may be explained by the difference in CSF Aβ_42_ cutoff values used (490 versus 603 pg/ml, respectively) or other patient-cohort specific differences. In 2010, Mulder et al. carried out a similar analysis of CSF Aβ_42_ and achieved a diagnostic sensitivity and specificity in between that of Kapaki and De Jong et al. (59). This may also be related to the fact that their chosen cutoff value (550 pg/ml) was also in between the two earlier cutoff values (Table 2).

**Table 2:** **Comparison of CSF biomarkers of AD in various studies**.

Reference	CSF biomarker	Disease comparison	No. of cases (AD/non-AD)	Threshold	Specificity (95% CI)	Sensitivity (95% CI)	AUC	PPV/NPV
Kapaki et al. (57)	Aβ1-42	AD versus HC	(49/49)	490 pg/ml	0.80 (0.66–0.90)	0.82 (0.68–0.91)	0.87	0.92/0.6
	Aβ1-42	AD versus NAND	(49/15)	435 pg/ml	0.80 (0.52–0.95)	0.71 (0.57–0.83)	0.76	0.84/0.65
De Jong et al. (58)	Aβ1-42	AD versus HC	(61/30)	603 pg/ml	0.93	0.93	N/A	0.97/0.88
Mulder et al. (59)	Aβ1-42	ProbAD versus HC	(131/248)	550 pg/ml	0.83 (0.76–0.89)	0.85	0.93	N/A
Vos et al. (28, 29)	Aβ1-42	AD versus naMCI	(39/226)	624 pg/ml	0.71 (0.57–0.85)	0.55 (0.33–0.77)	N/A	0.48/0.76
	Aβ1-42	AD versus aMCI	(132/399)	550 pg/ml	0.58 (0.57–0.85)	0.75 (0.64–0.87)	N/A	0.56/0.78
Kapaki et al. (57)	Total tau	AD versus HC	(49/49)	317 pg/ml	0.96 (0.86–0.99)	0.88 (0.75–0.95)	0.95	0.98/0.73
	Total tau	AD versus NAND	(49/15)	437 pg/ml	0.93 (0.68–0.99)	0.71 (0.57–0.83)	0.76	0.94/0.68
De Jong et al. (58)	Total tau	AD versus HC	(61/30)	352 pg/ml	0.97	0.79	N/A	0.98/0.69
Mulder et al. (59)	Total tau	AD versus HC	(131/248)	427 pg/ml	0.78 (0.65–0.91)	0.60 (0.39–0.81)	N/A	0.57/0.80
Vos et al. (28, 29)	Total tau	AD versus naMCI	(39/226)	427 pg/ml	0.78 (0.65–0.91)	0.60 (0.39–0.81)	N/A	0.57/0.80
	Total tau	AD versus aMCI	(132/399)	524 pg/ml	0.61 (0.50–0.72)	0.74 (0.62–0.85)	N/A	0.57/0.77
Kapaki et al. (57)	Aβ1-42/T-tau	AD versus HC	(49/49)	2.27	0.86 (0.73–0.94)	0.96 (0.86–0.99)	0.96	0.95/0.88
	Aβ1-42/T-tau	AD versus NAND	(49/15)	1.06	1.0 (0.78–1.0)	0.71 (0.57–0.83)	0.92	1.00/0.70
De Jong et al. (58)	Aβ1-42/T-tau	AD versus HC	(61/30)	1.895	0.95	0.97	N/A	0.98/0.91
Vos et al. (28, 29)	Aβ1-42/T-tau	AD versus naMCI	(39/226)	0.96	0.54 (0.38–0.69)	0.90 (0.77–1.00)	N/A	0.57/0.80
	Aβ1-42/T-tau	AD versus aMCI	(132/399)	0.78	0.38 (0.27–0.48)	0.98 (0.94–1.00)	N/A	0.57/0.77
Shaw et al. (60)	Aβ1-42; T-tau with APOE ε4	AD versus NC	(100/114)	0.34	0.80	0.98	0.94	0.86/0.97

*Aβ_42_, amyloid-beta1-42 peptide; AD, Alzheimer’s disease; aMCI, amnestic mild cognitive impairment; HC, healthy control; MCI, mild cognitive impairment; naMCI, non-amnestic mild cognitive impairment; NAND, non-AD neurodegenerative dementias; PPV/NPV, positive predictive value/negative predictive value; T-tau, total tau; CSF, cerebrospinal fluid*.

In a recent study reflecting the shift in Alzheimer’s biomarker research focus, Vos et al. did not seek to differentiate AD from healthy controls or NAND. Instead, they studied whether CSF Aβ_42_ could potentially predict if subjects with aMCI and naMCI would progress to AD (28). Recent studies have shown that AD pathology is common in subjects with naMCI; hence, Vos et al. investigated whether the diagnostic performance of this test would differ between these two types of MCI (61). With this CSF test, they were able to achieve slightly better diagnostic capability in predicting AD arising from aMCI compared to AD arising from naMCI, although the results were far from being optimal; these findings require independent validation. Although the same sandwich ELISA method (Innotest b-amyloid 1-42; Innogenetics, Ghent, Belgium) was used for CSF Aβ_42_ quantification in all of the above studies, there was variation in the CSF Aβ_42_ cutoff value used for diagnosis, and this may potentially account for the observed differences in diagnostic performance in the different studies. These results are summarized in Table 2.

Although CSF Aβ_42_ has been the most widely studied CSF Aβ peptide in AD, some studies have also investigated the biomarker potential of Aβ_40_, another component of the amyloid plaque that is primarily found in blood vessel walls (62). Gao et al. compared the diagnostic potential of the Aβ_40_ oligomer to that of the Aβ_42_ monomer and found Aβ_42_ monomers to be superior (63). With a chosen cutoff value of 150 pg/ml (Aβ_42_ monomers are reduced in the CSF of AD patients), Aβ_42_ monomers exhibited a diagnostic accuracy of 88% compared to 83% for Aβ_40_ oligomers when delineating between healthy controls and AD patients. An increasing number of studies have also found value in using the ratio of Aβ_42_ to Aβ_40_ as an AD biomarker. In 2015, Dumurgier et al. investigated the diagnostic potential of Aβ_42/_Aβ_40_ ratio in a multicenter study (64). Overall, the Aβ_42/_Aβ_40_ ratio and isolated Aβ_42_ levels were found to have similar diagnostic accuracy in terms of discriminating AD from non-AD subjects with a sensitivity/specificity of 0.73/0.78 and 0.78/0.79, respectively. Interestingly, the use of the Aβ_42/_Aβ_40_ ratio reduced the number of indeterminate CSF profiles by half and was in better agreement with the clinical diagnosis. These results raise the possibility that CSF Aβ_42/_Aβ_40_ ratios may better reflect brain amyloid production and warrant further investigation in longitudinal studies.

## CSF Tau Protein

Along with Aβ senile plaques, NFTs are also present within the hippocampus and cerebral cortex in the early stages of AD. These tangles are composed of filamentous hyperphosphorylated tau protein, whose total concentration is increased in the CSF of AD patients (10, 65). Total CSF tau levels have also been found to be increased in 90% of MCI patients who progressed to AD, implying that CSF tau protein may be a good biomarker for screening MCI patients who may eventually develop AD (65).

Kapaki et al. evaluated the diagnostic potential of total CSF tau protein as a biomarker of AD versus normal aging and other NAND within the Greek population (57). They observed a 3.5-fold increase in CSF t-tau levels in AD patients compared with healthy controls, whose basal level was 140 pg/ml. In 2006, De Jong et al. found a similar fold difference of 3.3 for CSF t-tau levels in their AD group compared to healthy controls (58). Mulder et al., using a higher cutoff value for CSF tau than De Jong and Kapaki et al., obtained a lower specificity value of 0.78, although the sensitivity remained high at 0.85 (59). In the same 2013 study cited earlier, Vos et al. also studied whether CSF t-tau protein could potentially predict if aMCI and naMCI would progress to AD (28) and found that CSF t-tau was equally predictive of AD development in both groups.

In addition to CSF t-tau, the main component of NFTs, CSF levels of phosphorylated tau (p-tau) have also been investigated as an AD biomarker. In 2006, De Jong et al. investigated the diagnostic potential of p-tau for delineating AD patients and healthy controls. With a cutoff value of 68 pg/ml, p-tau yielded a sensitivity of 0.75, specificity of 0.85, and PPV/NPV of 0.93/0.55. p-tau did not reach the diagnostic value of t-tau in this study. Similar findings were reported by Mulder et al. (59), where a cutoff value of 52 pg/ml for p-tau yielded a sensitivity of 0.85 and specificity of 0.68.

The same sandwich ELISA method (Innotest hTAU-Ag; Innogenetics, Ghent, Belgium) was used for all of the above studies investigating CSF t-tau and p-tau. Once again, there was variation in the chosen diagnostic test cutoff values in the different studies, and this could potentially account for the reported differences in the sensitivity and specificity metrics reported in these studies. These results are summarized in Table 2.

## CSF Aβ1-42/Total Tau Ratios

Interestingly, some of the above studies suggest that it is actually the ratio of these two CSF molecules that may serve as the ultimate biomarker (28, 66), since combining CSF Aβ1-42 and t-tau increased the diagnostic accuracy compared to when they were used alone. This may reflect a physiological relationship between Aβ1-42 and t-tau that is particularly related to AD pathogenesis (67). At best, CSF Aβ1-42/total tau ratios attained specificity, sensitivity, and PPV values exceeding 95% in distinguishing AD from healthy control. The NPV of this ratio was about 90% in distinguishing AD from healthy controls and 70% in distinguishing AD from disease controls (i.e., NAND) (Table 2).

## CSF Visinin-Like-Protein-1 (VILIP-1)

Visinin-like-protein-1 has been studied less extensively as a neurochemical biomarker compared to Aβ_42_ and tau protein. The family of visinin-like proteins, which are neuronal calcium sensor proteins, plays a role in both neuroprotective and neurotoxic functions and have been implicated in a number of neurodegenerative diseases (68, 69). In particular, VILIP-1 has been identified as a marker of neuronal injury through GWAS and brain injury models where it is released into the CSF from injured neurons (70). Tarawneh et al. (71) have reported that CSF VILIP-1 levels differ between AD and normal cognitive subjects (520 versus 396 pg/ml, respectively) and correlate with CSF t-tau, p-tau-181, and brain volumes in AD. CSF VILIP-1/Aβ_42_ ratios also differed significantly between AD and normal cognitive subjects (1.55 versus 0.74, respectively) and correlated with PiB-PET cortical binding potential, which is positively reflective of amyloid load. Clearly, further studies are warranted to compare the performance of this AD marker with previous biomarker candidates studied in the CSF. For reference, PiB-PET has been used in some studies in parallel with or to confirm CSF Aβ assay results since it is one of the leading neuroimaging tools with the potential to detect and provide quantitative measures of AD amyloid pathology *in vivo* at its early stages, with potential for longitudinal tracking (72, 73). The potential utility of imaging biomarkers in AD diagnostics have been reviewed recently (12, 14).

## CSF YKL-40

Although markers associated with the underlying neuropathology in AD, namely CSF Aβ and CSF tau, have remained the top candidates for AD diagnostics, these markers have also been found to be altered in other disorders such as dementia with Lewy bodies (DLB), frontotemporal dementia (FTD), and vascular dementia (VAD). As a result, there has been a concerted effort to identify other biomarkers that can potentially distinguish these different neurological disorders from AD (74). In 2010, Craig-Schapiro reported elevated levels of CSF YKL-40 among very mild and mild-type AD dementia subjects using ELISA (75). YKL-40, also known as chitinase-3 like-1, is upregulated in various inflammatory conditions and expressed by different cell types such as activated neutrophils, macrophages, chondrocytes, and vascular smooth muscle cells (76). In the brain, it is mainly expressed by astrocytes. Further, in 2015, Wennström et al. reported that CSF YKL-40 was elevated in AD subjects, but not in those with Parkinson’s disease or DLB, thus making it a potential distinguishing biomarker between these neurological disorders (77). Hellwig et al. found that YKL-40 had an AUC of 0.74 in distinguishing AD from non-AD subjects (78). However, Janelidze et al. reported no improvement in diagnostic accuracy of either prodromal AD or AD dementia when using YKL-40, compared to using core CSF AD biomarkers (Aβ and tau), although YKL-40 was selectively increased in AD and FTD patients compared to the other dementias including DLB, VAD, and Parkinson’s disease dementia (79). There is no doubt that future studies will investigate the diagnostic potential of YKL-40 further and confirm if YKL-40 is clinically useful in distinguishing between the different dementias.

## CSF Neurogranin (NGRN)

Another newly reported biomarker candidate is NGRN, a postsynaptic protein expressed mostly in the cortical areas of the brain suggesting a connection to cognition. It is concentrated in the dendritic spines of principal excitatory synapses, whose translocation thereof is impaired in AD; decreased NGRN levels have been observed in the hippocampus and cortex (80). In 2015, Kester et al. reported that CSF NGRN levels were higher in MCI and AD subjects, compared to cognitively normal subjects (81). This relationship persisted upon follow-up, 3.8 years later. It is believed that the increased levels of CSF NGRN may be secondary to their impaired translocation to dendritic spines. In the same study by Hellwig et al. discussed above, CSF NGRN was found to have an AUC value of 0.85 when distinguishing AD from non-AD dementia subjects, suggesting that it may be more valuable diagnostically than YKL-40, and that it may also have the potential to distinguish different neuronal disorders, just like YKL-40 (78). In the same study by Janelidze discussed above, no improvement in diagnostic accuracy was noted in assessing prodromal AD or AD dementia when using CSF NGRN, compared to using the core CSF AD biomarkers (79). The extent to which CSF NGRN, CSF YKL-40, CSF tau, CSF Aβ1-42, and other potential CSF proteins correlate with each other remains unclear. Clearly, this insight would be significant in constructing future diagnostic panels.

## NGRN/BACE1 Ratio

Other studies have shown that combining CSF biomarkers have improved diagnostic performance, as that seen with CSF Aβ1-42/tau ratios. In 2016, De Vos et al. investigated NGRN/BACE1 ratio as a potential AD biomarker and found that it correlated with yearly decline in mini-mental state examination (MMSE) scores in patients with MCI and dementia due to AD (β = −0.018 and −0.051, respectively) (82). β-site amyloid precursor protein cleaving enzyme 1 (BACE1) is the β-secretase enzyme required for the production of the neurotoxic Aβ peptide and is thus considered to have a critical early role in the etiology of AD (83). Although extensive diagnostic values are lacking, this study showed this ratio’s potential as a prognostic biomarker and will undoubtedly be studied further for possible integration into clinical trials.

## CSF Aβ1-42, Total Tau with *APOE* ε*4*

Combining both CSF proteins and genetic biomarkers in 2009, Shaw et al. developed a logistic regression model based on CSF Aβ_42_, t-tau, and the *APOE* ε*4* allele (60). Logistic regression analyses were performed using sex, years of education, age at the time of lumbar puncture, *APOE* ε4 allele gene dosage (none, heterozygous, or homozygous), and each of the three CSF biomarkers: Aβ42, t-tau, and phosphorylated tau (p-tau-181), with backward elimination and insertion into the model that had only Aβ_42_ and t-tau as variables, in order to identify optimal markers. With these multiple markers, they achieved a specificity of 79.5%, a sensitivity of 98.2%, an ROC AUC value of 94.2%, and a PPV/NPV of 85.7/97.2% (Table 2). These sensitivity, NPV, and ROC AUC values were certainly among the highest reported, compared to the performance of the individual CSF biomarkers. However, the associated specificity was closer to the average specificities reported previously, where CSF t-tau, as a single biomarker, had a specificity of 96%. One potential scenario could be to use the combined biomarker panel for initial screening (as it has high sensitivity) followed up by individual biomarker assays that have been shown to have higher specificity metrics.

## Blood-Derived Biomarkers

In addition to studying CSF biomarkers, researchers have sought less invasive sources, such as blood (10, 84). Obtaining blood samples is relatively painless and inexpensive, giving potential blood-based biomarkers further advantage over the CSF-based markers. Some of the blood biomarkers appear to be just as diagnostically accurate as the CSF-based and genetic biomarkers though further validation is warranted. The major blood-derived biomarkers for AD discussed here have been identified through proteomic, lipidomic, and genomic profiling.

## Discovery-Based Plasma Protein Panels

Ray et al. investigated biomarkers that could predict the progression of MCI to AD so that early preventative treatment can be delivered to AD-presymptomatic patients (85, 86). Using a filter-based, arrayed sandwich ELISA analysis of plasma from presymptomatic to late state AD and non-demented controls, they were able to identify a large number of elevated proteins within these subjects. Using predictive analysis of microarrays, Ray et al. further narrowed down potential candidates to a predictor panel of 18 proteins (86). With this 18-protein panel, they were able to achieve a diagnostic accuracy of 90% in distinguishing AD and MCI subjects, where 81% of MCI subjects were accurately identified to progress to AD after a 2- to 6-year follow-up period (Table 3). They were classified with 90% positive agreement for the AD samples and 88% negative agreement for the non-AD samples. Although published in 2007, these proteins have not been replicated widely, while one study has reported no difference in a majority of these 18 proteins between AD and healthy subjects (85). In 2014, in an independent study, Hye et al. achieved a high diagnostic accuracy with a 10-protein panel (87). Of these proteins, ICAM-1 was the only protein that overlapped with Ray et al.’s 18-protein panel.

**Table 3:** **Comparison of blood biomarkers of AD in various studies**.

Reference	Blood biomarker	Disease comparison	No. of cases (AD/non-AD)	Accuracy	Specificity (95% CI)	Sensitivity (95% CI)	AUC	PPV/NPV
Ray et al. (86)	18-protein panel	AD versus MCI	(22/17)	0.90	N/A	N/A	N/A	N/A
Hye et al. (87)	10-protein panel with *APOE* ε*4*	AD versus MCI	(476/220)	0.87	0.88	0.85	0.84	0.69/0.95
Kiddle et al. (88)	Covariates^a^	AD versus HC	(80/53)	0.71	0.74	0.70	N/A	0.89/0.45
	Replicated proteins with covariates^a^	AD versus HC	(80/53)	0.77	0.72	0.80	N/A	0.83/0.68
Mapstone et al. (89)	10 metabolite panel	aMCI/AD versus HC	(21/20)	N/A	N/A	N/A	0.77	N/A
	10 metabolite panel	Converters_pre_ versus HC	(10/20)	N/A	0.90	0.90	0.92	N/A
Leidinger et al. (90)	12-miRNA signature	AD versus HC	(48/22)	0.933 ± 0.046 (0.924–0.942)	0.951 ± 0.054 (0.941–0.962)	0.915 ± 0.058 (0.904–0.927)	N/A	0.949/0.918
	12-miRNA signature	AD versus MCI	(94/18)	0.756 ± 0.078 (0.741–0.772)	0.767 ± 0.083 (0.751–0.784)	0.746 ± 0.097 (0.727–0.765)	N/A	0.779/0.724
Bhatnagar et al. (91)	miRNA-34c	AD versus HC	(25/27)	0.94	0.96	0.92	0.99	0.958/0.923
Cheng et al. (92)	16 miRNA signatures with *APOE* ε*4*	AD versus HC	(16/36)	N/A	0.77	0.87	N/A	N/A

*AD, Alzheimer’s disease; aMCI, amnestic mild cognitive impairment; APOE, apolipoprotein E; Converters_pre_, phenoconverters prior to conversion; HC, healthy control; MCI, mild cognitive impairment; naMCI, non-amnestic mild cognitive impairment; NAND, non-AD neurodegenerative dementias; PPV/NPV, positive predictive value/negative predictive value; miRNA, microRNA*.

*^a^The covariates examined included age at disease onset, gender, and presence of APOE ε4 allele*.

In their 2006 study, Hye et al. performed proteomic analysis of plasma samples from AD and healthy elderly subjects using isoelectric focusing followed by polyacrylamide gel electrophoresis (93). A set of 15 proteins was identified and validated using western blotting. For diagnostic analysis, the profile of these 15 proteins in AD and healthy elderly subjects were measured using 2-D gel electrophoresis (2-DGE). Further support vector machine analysis of the 2-DGE data yielded a sensitivity of 56% and specificity of 80% for AD diagnosis (93).

In a later study, they analyzed plasma samples from AD, MCI, and elderly non-demented subjects using multiplex bead assays (Luminex xMAP) incorporated in 7 MILLIPLIEX MAP panels (87). In particular, Hye et al. focused on 26 proteins that had been previously identified as potential AD biomarkers. These biomarkers include proteins from their earlier study in 2006 [ceruloplasmin, complement factor H (CFH), and serum amyloid P-component precursor (SAP)] and proteins from the 18-protein panel reported by Ray et al. (93). Out of the 26 plasma proteins tested, Hye et al. found only 2 proteins, ApoE and CFH, to be significantly different between AD and controls.

In this same study, Hye et al. then proceeded to identify plasma proteins that could predict the progression of MCI to AD, which was assessed based on the degree of hippocampal atrophy. Using multivariate linear regression analysis, they identified six proteins that predicted 19.5% of hippocampal volume loss in subjects with MCI: CLU, neuron-specific enolase (NSE), TTR, vascular adhesion molecule 1, and SAP. In addition, seven proteins were identified that predicted 11.9% of hippocampal volume loss in subjects with AD: APOA1, alpha-1 antitrypsin (A1AT), ApoC3, brain-derived neurotrophic factor, AB40, plasminogen activator inhibitor-1, and NSE. They reasoned that since these proteins reflected pathological load, they might also predict conversion from pre-disease states (i.e., MCI) to clinical dementia (i.e., AD). Applying the Naïve Bayes simple machine learning approach to a test set, they found that the average time of conversion of MCI to AD was approximately 1 year where a combination of 10 plasma proteins (TTR, CLU, cystatin C, A1AcidG, ICAM-1, CC4, pigment epithelium-derived factor, A1AT, RANTES, and ApoC3) coupled with the *APOE* ε*4* genotype yielded the greatest predictive potential with 85% sensitivity, 88% specificity, 0.87% accuracy, and 68.8/95% PPV/NPV (Table 3). This protein panel did not quite reach the 90% diagnostic accuracy reported in Ray et al.’s study, but one can certainly envision further fine tuning of these protein panels to attain better diagnostic performance in future studies.

## Protein Panel Combined with *APOE* ε*4* Allele

In 2014, Kiddle et al. reviewed 21 discovery or panel-based proteomic studies aimed at identifying protein biomarkers in AD. Four of these markers (α-1 antitrypsin, α-2-macroglobulin, APOE, and complement C3) were replicated in 5 of the 21 independent studies despite the use of different methodologies (88). Certain covariates, such as the age of disease onset and gender, coupled with the presence of *APOE* ε*4* allele had some predictive capability (Table 3). Combining these covariates with these four most highly replicated blood protein biomarkers improved the predictive potential (Table 3). These results indicate that combining biomarker leads from multiple OMICs approaches (e.g., proteomics and genomics) may lead to AD biomarkers with improved predictive performance. The biomarkers used in these studies also represent the molecules that have been validated in the largest numbers of independent patient cohorts.

## Discovery-Based Blood Lipid Panels

Recently Mapstone et al. reported a set of 10 phospholipids from peripheral blood that predicted phenoconversion to either aMCI or AD within 2–3 years, with over 90% accuracy (89). These researchers performed an untargeted metabolomic analysis of 124 plasma samples from aMCI/AD (including post-phenoconverters) that were taken at the start of the study, and 3 years later. They then used tandem mass spectrometry to identify 10 metabolites that constituted a discriminatory signature: phosphatidylcholines (PCs) [PC diacyl (aa) C36:6, PC aa C38:0, PC aa C38:6, PC aa C40:1, PC aa, C40:2, PC aa C40:6, PC acyl–alkyl (ae) C40:6], lysophosphatidylcholine (lysoPC a C18:2), and acylcarnitines (ACs) [propionyl AC (C3) and C16:1-OH]. These 10 lipids were reduced in the plasma of the Converter_pre_ group compared to the healthy controls, and they remained so even after phenoconversion to aMCI/AD (Converters_post_) (Table 3). This 10-lipid panel still needs to be validated in independent studies. Also, the biological relevance of these lipids to the pathogenesis of AD warrants careful evaluation.

## Discovery-Based Blood microRNA (miRNA) Signatures

Several groups have attempted to identify potential miRNA signatures for AD (94, 95). One of the highest diagnostic accuracies among these studies was reported by Leidinger et al., who identified a panel of 12 miRNAs through NGS of blood miRNA transcriptomes from AD patients, MCI patients, and healthy controls (90). This 12-miRNA signature was validated by RT-qPCR in a group of 202 patients suffering from other neurological disorders, which included MCI. While some blood miRNA were reduced in AD (hsa-let-7f-5p, hsa-miR-1285-5p, hsa-miR-107, hsa-miR-103a-3p, hsa-miR-26b-Sp, hsa-miR-26a-Sp, and hsa-miR-532-Sp), others were elevated (hsa-miR-151a-3p, brain-mir-161, hsa-let-7d-3p, brain-miR-112, and hsa-miR-5010-3p), when compared to the healthy controls. Leidinger et al. were able to differentiate between AD and controls with an accuracy of 93%, a specificity of 95%, and a sensitivity of 92%, while the differentiation of AD from other neurological diseases (MCI, Parkinson’s disease, multiple sclerosis, and major depression) attained accuracies between 74 and 78% (Table 3). Though these results are promising, further independent validation is warranted.

In 2014, Bhatnagar et al. reported that miRNA-34c is more highly expressed in the plasma (0.29 versus 0.1) and peripheral blood mononuclear cells (0.08 versus 0.07) of AD patients compared to healthy controls (91). They then analyzed whether blood miRNA-34c could distinguish AD subjects from age-matched healthy counterparts. They achieved relatively high diagnostic accuracies, compared to other AD miRNA biomarker studies, with a sensitivity of 0.92, a specificity of 0.96, and a PPV/NPV of 0.958/0.923. Validation in independent cohorts and comparison to other neurological disease controls are warranted. Interestingly, earlier studies have shown that miRNA-34c plays a role in the repression of genes involved in cell survival/apoptosis and neuroprotective signaling (96, 97).

## miRNA Signature Combined with *APOE* ε*4* Allele

In 2014, Cheng et al. identified a set of 16 miRNAs in the plasma of AD that could serve as a potential disease signature (92). This 16 miRNA signature was created using NGS with qRT-PCR validation, a method used previously by Leidinger et al., though, there was no overlap between the miRNA signatures uncovered in these two studies (90). While some miRNA were reduced in AD (hsa-miR-1306-5p, hsa-miR-342-3p, and 15b-3p), others were elevated (hsa-miR-361-5p, hsa-miR-30e-5p, hsa-miR-93-5p, hsa-miR-15a-5p, hsa-miR-143-3p, hsa-miR-335-5p, hsa-miR-106b-5p, hsa-miR-101-3p, hsa-miR-424-5p, hsa-miR-106a-5p, hsa-miR-18b-5p, hsa-miR-3065-5p, hsa-miR-20a-5p, and hsa-miR-582-5p), when compared to the healthy control subjects. Combining this deregulated 16 miRNA signature with the presence of the *APOE* ε*4* allele, Cheng et al. were able to discriminate AD subjects from healthy subjects with 77% specificity and 87% sensitivity (Table 3). The diagnostic parameters of this study may be less optimal than that of Leidinger et al. (77% specificity and 87% sensitivity versus 95% specificity and 92% sensitivity, respectively), even with the integration of the *APOE* ε*4* allele, possibly due to a smaller cohort size (*n* = 60 versus *n* = 202, respectively). All of the above miRNA studies warrant independent validation and analysis in longitudinal data sets so as to identify common, reproducible themes. This becomes particularly pertinent given that there was no overlap between the findings of these two reports.

## Plasma Aβ1-42/Aβ1-40 Ratios

Several studies have examined the predictive ability of plasma Aβ levels. This is not surprising given that CSF Aβ levels and Aβ accumulation, as determined by PiB-PET, are validated AD biomarkers (5, 7, 8, 29, 98). However, recent findings on the relationship between AD pathogenesis and plasma Aβ levels have been contradictory. Whereas some argue that an increase in the plasma Aβ_42_/Aβ_40_ ratio is related to an increased risk of developing AD (99), others report that it is actually the reduction of plasma Aβ_42_/Aβ_40_ ratio that increases risk (72, 100–102). Interestingly, positron emission tomography (PET) measures of brain amyloid burden does show an association between reduced plasma Aβ_42_/Aβ_40_ and increased brain amyloid load (72, 102). In addition, a recent study by Chouraki et al., examining the levels of plasma Aβ_42_ and Aβ_40_ in 2,189 dementia-free individuals over an 8-year period, found that lower levels of plasma Aβ_42_/Aβ_40_ ratio were associated with an increased risk of developing dementia or incident AD (100). Likewise, Fei et al. followed the plasma Aβ_42_/Aβ_40_ ratios of 588 subjects with MCI over 4–6 years to ascertain if these ratios can be used to identify those who may convert to AD. Fei et al. reported that plasma Aβ_42_/Aβ_40_ ratios exhibited a sensitivity of 85.7% and specificity of 69.7% in this respect (103). Although the diagnostic value of plasma Aβ_42_/Aβ_40_ ratios did not quite surpass that of CSF Aβ_42_ used alone, these results call for further investigation of plasma biomarkers that may be reflective of amyloid load.

## Blood Biomarkers of Neocortical Amyloid Burden (NAB) in AD

Along the same vein, researchers have searched for other blood-based biomarkers that may reflect NAB, a pathophysiology known to increase the risk of AD (7, 104, 105). With these particular NAB biomarkers, researchers hope to identify an optimal window of treatment with anti-Aβ therapies (106). Very recently, Ashton et al. reported that a single blood protein, fibrinogen γ-chain (FGG), selected from 17 discovery candidates, predicted high NAB when combined with age, yielding a sensitivity of 59% and specificity of 78%. High NAB was considered for standardized uptake value ratios (SUVR) greater than 1.3 in PiB-PET scans, a cutoff value supported by previous studies (105). Other studies argue that an SUVR greater than 1.5 is a more appropriate cut off, but whether this difference has a significant impact on diagnostic performance is yet to be determined (107). Whereas plasma Aβ_42_/Aβ_40_ ratios exhibit moderately high sensitivity and moderately low specificity, FGG exhibits the inverse diagnostic relationship (104).

Voyle et al. also noted the promise of FGG. Very recently, they constructed a 5-metabolic feature panel, further enriched by the addition of FGG, to identify those with high NAB. These 5-metabolic features were selected from an initial number of 3,196, after rigorous metabolomic analysis using multiple metabolic feature models (108). However, only four of the five metabolites, phosphatidylcholine (PCaa 36:6), PE 39:7, anandamide, and anandamide isotope, were putatively identified. When combined with FGG, this 4-plex metabolic panel identified high-NAB subjects with a sensitivity of 71%, specificity of 84%, and accuracy of 79%, diagnostic values that exceed that of previously reported blood-based biomarker for NAB. Collectively, these studies indicate that researchers are close to identifying blood-based biomarkers of NAB with accuracies approaching 80%.

## Longitudinal Studies

Recent studies have shown that approximately 30% of age-matched HC individuals have preclinical AD based on two currently validated biomarkers, Aβ in PiB-PET measurements or Aβ in the CSF (6, 8, 98). This frequency does differ between reports, likely due to methodological differences between studies (109). These results and results from other clinicopathological and biomarker studies support the existence of a long preclinical stage during which AD pathologies develop, preceding the appearance of cognitive symptoms. Because of this, the primary focus of Alzheimer’s research has shifted from differentiation between HC and AD to determining the rates of cognitive decline in subjects who have preclinical, prodromal, or clinical AD. Through longitudinal studies, researchers envision that an effective treatment window may emerge, which could potentially permit therapeutic intervention, including anti-Aβ therapies (106).

## Baseline CSF Biomarkers Versus Long-Term Cognitive Decline

Based on their promising results in 2011, Tarawneh et al. examined the long-term prognostic potential of CSF VILIP-1 and VILIP-1/Aβ_42_ ratios over a period of 2.6 years among AD patients (71, 110). CSF was only collected once at the beginning of the study and analyzed for t-tau, p-tau (or p-tau-181), Aβ_42_, and VILIP-1. To assess the progression of cognitive decline, 60 AD subjects were cognitively assessed using the clinical dementia rating sum of boxes (CDR-SB), annually, with an average of 3 cognitive assessments per subject. The CDR has high inter-rater reliability, is sensitive to clinical progression, and is highly predictive (93%) of autopsy-confirmed AD (111, 112). Once AD diagnosis using CDR-SB was confirmed (using a cut off of 0.5), a psychometric test battery assessing a broad spectrum of cognitive functions was administered to all subjects. This test quantified the episodic memory composite, the semantic memory composite, the working memory composite, the visual spatial composite, and the global psychometric composite of all subjects.

Not surprisingly, higher basal levels of all the CSF biomarkers examined were associated with an increased rate of decline. However, CSF VILIP-1 and p-tau-181 proteins predicted the greatest decline (1.61 and 1.583, respectively) within the upper tercile group (with cut offs at 560 and 93 pg/ml, respectively), while in the lower tercile group, CSF VILIP-1 and t-tau protein predicted the greatest decline (0.852 and 0.828, respectively) of CDR-SB. For CSF VILIP-1/Aβ_42_ ratios, the rate of CDR-SB decline for both the upper and lower tercile group were the lowest among all the CSF biomarkers assessed, although still discernable. When analyzing the rate of decline for global psychometric and episodic memory composite scores, CSF VILIP-1/Aβ_42_ ranked highest (0.615 and 0.674, respectively), followed by CSF p-tau-181/Aβ_42_ (0.594 and 0.659, respectively). This study’s conclusion that CSF VILIP-1 and CSF VILIP-1/Aβ_42_ ratio can predict global cognitive changes resonates well with findings from other studies, as described below.

## Baseline Disease Stage Versus Long-Term Cognitive Decline

Vos et al. investigated the prevalence and long-term outcome of preclinical AD based on new classification criteria employing preclinical disease stages, where stage 1 subjects were cognitively normal individuals with abnormal amyloid markers, stage 2 subjects had abnormal amyloid and neuronal injury markers with no subtle cognitive changes, and stage 3 subjects had abnormal amyloid and neuronal injury markers with subtle cognitive changes (29). Three hundred eleven subjects underwent annual cognitive assessment, which included CDR and CDR-SB, MMSE, and a psychometric test battery. At the beginning of the study, all groups, including preclinical AD, were considered to have a CDR of 0, meaning that no dementia was present. Vos et al. sought to map their progression to a CDR ≥ 0.5 (onset of symptomatic AD) over a 14-year period. CSF samples were obtained from all subjects and analyzed for t-tau, p-tau-181, and Aβ_42_ levels. *APOE* ε*4* allele presence was also recorded, with stage 3 subjects having the largest prevalence of *APOE* ε*4* positivity (69%). Indeed, stage 3 subjects showed the highest rate of progression to clinical dementia (56% after 5 years), followed by stage 2 (26%), stage 1 (11%), and the normal subjects (2%). This increased rate of progression to clinical dementia with increased preclinical stage score was also reflected by their progressively increasing deficits of CSF Aβ_42_ levels (355, 350, 321 pg/ml for stages 1, 2, and 3, respectively). These studies underscore the importance of factoring in baseline clinical symptoms, laboratory markers, and genotype in order to prognosticate future disease progression. Such a multi-pronged panel could also be of immense utility in tracking treatment response in future clinical trials.

## CSF Biomarker Levels Versus EOAD Progression

In 2014, Fagan et al. performed a longitudinal investigation of preclinical biomarkers based on clinicopathological evidence suggesting that the early pathological events of AD occur years before the onset of cognitive symptoms (8, 113). This study differed significantly from the other studies discussed above in that they studied subjects who were autosomal-dominant for AD and thus had EOAD in contrast to LOAD.

In their follow-up study, they enlisted a large cohort of 146 mutation carriers (MCs) and 96 mutation non-carriers (NCs) from the dominantly inherited Alzheimer network, spanning a wide range of estimated number of years to symptom onset. They analyzed longitudinal CSF samples from a subset of 37 individuals (11 NCs and 26 MCs) examined every 5 years and found that in asymptomatic MCs, the serial change in CSF biomarkers was similar to those seen in previous cross-sectional LOAD studies, i.e., elevations in CSF t-tau, p-tau-181, and VILIP-1 and reduction in CSF Aβ_42_. The observation of CSF VILIP-1 elevations in MCs at least 15 years before their estimated age at symptom onset (EAO), with concentrations being even higher in individuals who were closer to their EAO, suggests a robust phase of neuronal injury and/or death that begins before the onset of cognitive symptoms. However, once the MCs reached their age of AD onset, Fagan et al. observed a decrease in CSF markers of neuronal injury/death (i.e., CSF t-tau, CSF p-tau-181, and VILIP-1) as time progressed. The rate of longitudinal change (adjusted for gender and *APOE* ε*4* genotype) was documented as +6.90 to −10.79 pg/ml per year for CSF tau, 1.34 to −6.62 pg/ml per year for CSF p-tau-181, and −0.903 to −14.60 pg/ml per year for VILIP-1. This longitudinal evolution suggests that once these neuronal injury markers reach their peak release, which may occur during age at symptom onset, the rate of release of these markers may then slow down, in line with a progressive decline in neuronal injury/death. This study provides an impetus for further longitudinal studies of LOAD, with systematic serial monitoring of not only CSF markers, but also blood-derived biomarkers.

## Combined GRS Versus Risk of Conversion/Rapid Progression to MCI

In 2013, Rodríguez-Rodríguez et al. evaluated whether a combined GRS, including *APOE, BIN1, PICALM*, and *CLU*, is associated with either risk of conversion or with rapid progression from MCI to AD (114). They followed 288 subjects with MCI over a mean period of 26.3 months and identified 118 MCI-converters to AD and 170 MCI-non-converters. Perhaps not surprisingly, *APOE e4* was significantly associated with conversion risk (OR = 4.63) and rapid progression (HR 1.77), while CLU was associated with decreased conversion risk (OR 0.25), which is in agreement with previous studies suggesting that it has a neuroprotective role in AD pathogenesis (41). In contrast, Rodríguez-Rodríguez et al. found no association between the combined GRS and the risk of conversion from MCI to AD. However, they did find that those who did convert and carried six risk alleles or more progressed about twice as quickly to AD than those who carried less than six risk alleles, with the acceleration of progression being an average of 5 months. This is somewhat of an improvement compared to using *APOE e4* alone (HR 1.77). Unfortunately, the correlation between GRS (115, 116) and severity of AD development was not explored. Studies of this nature, which factor in multiple biomarkers, including the GRS, CSF, and blood-based biomarkers, will be useful for defining disease progression with better accuracy and possibly for designing interventional therapies.

## Clinical Trials

There has been an increasing push to evaluate potential AD biomarkers not only for diagnostic accuracy in the preclinical stages of AD but also in their ability to serve as prognostic markers and theranostic markers of response to AD treatment (117). As mentioned previously, disease-modifying treatments are most likely to have maximal benefit during the preclinical stages of AD; hence, the focus of drug development has shifted from the dementia stage of the disease where previous treatment methods were found to yield only modest if any benefit (118, 119). Compared to the wealth of available biomarkers, very few of these have been factored into clinical trials. Where biomarkers have been utilized in clinical drug studies, they have served as inclusion criteria for AD pathology presence and trackers of biological effects of treatment. Out of these studies, the bapineuzumab and solanezumab studies, which utilized PiB-PET and CSF biomarkers, initially showed promising results and progressed to phase III trials, where they ultimately failed (115, 116, 120, 121). Despite these negative results, biomarkers are clearly valuable tools in clinical trials, as is becoming evident from lessons learned in other fields. The bottleneck is in deciding what the optimal biomarkers to use are. With this in mind, there has been a call for more longitudinal studies on biomarker trajectories, linking neuropathology to biomarkers, and discovering novel biomarkers reflecting other disease processes downstream of initial AD pathology (117). Undoubtedly, the use of the most promising CSF and blood biomarkers arising from these studies, alongside with concurrent neuroimaging biomarkers, is likely to play an increasingly important role in future clinical trials.

## Conclusion

A clinically useful biomarker should preferentially have a sensitivity, specificity, PPV, and NPV exceeding 90%. Using these criteria, several biomarkers discussed above, including CSF Aβ1-42/t-tau ratio (Table 2), 12-miRNA signature (Table 3), and miRNA-34c (Table 3), look promising as all of their diagnostic parameters exceed 90%. In addition, CSF Aβ_42_ and the blood-based 10 lipid test have both yielded reasonable sensitivity and specificity values, with Aβ_42_ alone yielding a PPV, at or greater than 90% (Tables 1 and 2, respectively). These biomarkers, particularly in combination, warrant further validation in multiple independent patient cohorts. Although initial results are promising, miRNA biomarkers in particular will need subsequent replication studies since this biomarker approach is newer. More importantly, efforts are warranted to mine newer biomarkers using more advanced screening platforms, including those that allow for global scans of proteins, peptides, and metabolites, in blood as well as CSF. With accelerated research, one is hopeful that improved, easily measurable biomarkers that can predict the rates of cognitive decline and NAB in subjects who have preclinical, prodromal, or clinical AD will emerge.

## Author Contributions

RH contributed to the drafting of the manuscript. CM worked on the organization, direction, and editing of the manuscript.

## Conflict of Interest Statement

The authors declare that the research was conducted in the absence of any commercial or financial relationships that could be construed as a potential conflict of interest.

## Abbreviations

AAO, age at symptom onset; Aβ_42_, amyloid-beta1-42 peptide; ACs, acylcarnitines; AD, Alzheimer’s disease; ADAD, autosomal-dominant for AD; A1AT, alpha-1 antitrypsin; aMCI, amnestic mild cognitive impairment; AβPP, amyloid-β precursor protein; APOE/ApoE, apolipoprotein E; BACE1, β-site amyloid precursor protein cleaving enzyme; BBB, blood–brain barrier; BIN1, bridging integrator 1; BDNF, brain-derived neurotrophic factor; CDR-SB, clinical dementia rating sum of boxes; CLU, clusterin; Converters_pre_, phenoconverters prior to conversion; CNS, central nervous system; CP, ceruloplasmin; CFH, complement factor H; CR1, complement receptor 1; CSF, cerebrospinal fluid; DIAN, dominantly inherited Alzheimer network; DLB, dementia with Lewy bodies; EAO, estimated age at symptom onset; EOAD, early onset Alzheimer’s disease; EYOs, estimated number of years to symptom onset; FGG, fibrinogen γ-chain; FTD, frontotemporal dementia. GRS; Genetic Risk Score; GWAS, genome-wide association studies; HC, healthy control; HuGENet, human genome epidemiology network; LOAD, late onset Alzheimer’s disease; naMCI, non-amnestic mild cognitive impairment; MMSE, mini-mental state examination; MCs, mutation carriers; NAND, non-AD neurodegenerative dementias; NFTs, neurofibrillary tangles; NGRN, neurogranin; NSE, neuron-specific enolase; NGS, next-generation sequencing; NCs, non-carriers; OR, odds ratio; PICALM, phosphatidylinositol clathrin assembly lymphoid-myeloid; PAGE, polyacrylamide gel electrophoresis; p-tau, phosphorylated tau; PAI, plasminogen activator inhibitor; PAM, predictive analysis of microarrays; PEDF, pigment epithelium-derived factor; PET, positive emission tomography; PC, phosphatidylcholine; PDD, Parkinson’s disease dementia; PPV/NPV, positive predictive value/negative predictive value; PSEN, presenilin; SAP, serum amyloid P-component precursor; SUVR, standardized uptake value ratio; SVM, support vector machine; TREM2, triggering receptor expressed on myeloid cells 2; T-Tau, total tau; VAD, vascular dementia; VCAM, vascular adhesion molecule; VILIP, visinin-like-protein; 2-DGE, 2-D gel electrophoresis.
